# Research on the influence mechanism of food packaging on consumers' behavioral intention to refuse food waste based on behavioral reasoning theory

**DOI:** 10.3389/fpsyg.2025.1630861

**Published:** 2025-09-24

**Authors:** Juncheng Mu, Linglin Zhou, Chun Yang

**Affiliations:** ^1^School of Fine Arts, Nanjing Normal University, Nanjing, China; ^2^School of Design, Jiangnan University, Wuxi, China

**Keywords:** sustainable consumption, food packaging, food wastage, behavioral intention, BRT

## Abstract

Given the escalating global concern regarding food waste, food packaging emerges as a critical area for intervention. Existing research, however, predominantly concentrates on consumer attitudes, offering limited insight into the role of food packaging in mitigating food waste. To address this gap, this study employs the BRT model, incorporating the variable of behavioral rationality into traditional theory and integrating values as a preceding variable. It integrates consumer values, behavioral rationality, attitudes, and behavioral intentions to explore the influence mechanism of food packaging on consumer behavior, explaining behavioral intentions through supporting/rejecting reasons. The study investigates how visual attributes, functionality, environmental sustainability, and social attributes within supporting reasons positively influence consumers' attitudes and behavioral intentions to reject food waste. Conversely, it investigates how information overload, functional deficiencies, inappropriate sizing, and high cognitive load within rejection reasons negatively impact these attitudes and intentions. By dissecting the positive and negative impacts of food packaging attributes on consumer attitudes and behavioral intentions across supporting and rejecting reasons, this research aims to identify key design factors for food packaging. This will fill existing research gaps, provide a theoretical foundation for food packaging design, and encourage consumers to adopt positive behaviors that reduce food waste. The findings indicate that: (1) consumer values significantly influence different behavioral attitudes; (2) supporting reasons related to food packaging positively impact the rejection of food waste behavior, while rejecting reasons have the opposite effect; (3) both positive and negative reasons significantly influence consumer attitudes and behavioral intentions, highlighting the need to optimize design elements in food packaging to positively influence consumer behavior; (4) packaging design factors have a more significant impact on intentions to conserve food than consumer attitudes themselves. This study extends the Behavioral Reasoning Theory (BRT) to understand food packaging's influence on consumer behavior, addressing contextual factors within BRT. Furthermore, it provides a scientific basis for food companies to optimize packaging design, which has practical implications for mitigating global food waste.

## Introduction

Food is a fundamental condition for human survival and an essential guarantee for the development of human society. However, the phenomenon of food waste is increasingly prevalent in our rapidly developing modern society. Food waste, as a significant and practical issue of global concern, has a considerable impact on food security and supply (Campoy-Muñoz et al., 2021). It is a widespread issue worldwide, with its definition evolving alongside the complexities of the food supply chain (Parfitt et al., 2010). According to estimates by the Food and Agriculture Organization of the United Nations (FAO), approximately one-quarter to one-third of food produced globally is wasted (Bellemare et al., 2017), with variations across different countries and regions. In developed areas, consumer-level food waste constitutes the largest proportion (Heng and House, 2022), while in developing regions, post-harvest losses are more significant (Parfitt et al., 2010). Due to conflicts in certain countries and regions, global food production is projected to decline in 2024, while the number of people suffering from hunger continues to rise, exceeding 860 million, posing a severe threat to global food security (Moffat, 1992). The FAO reports that the world is facing the most severe food crisis in 50 years (Wu et al., 2014). Consequently, the increasingly dire food crisis has led countries worldwide to recognize the importance of food security, attempting to alleviate and improve the issue of food waste through various methods (Boliko, 2019). Food waste is one of the critical causes of the food crisis, referring to food that is still usable but is lost at the retail or sales level due to consumer behavior, such as purchasing excessive amounts of food, discarding food before it is consumed (Zeineddine et al., 2021), or rejecting food that does not meet aesthetic standards (Bauer et al., 2023). These behaviors are often intentional acts of food waste (Dusoruth and Peterson, 2020). The severity of food waste behavior is particularly pronounced in countries and regions with high levels of urbanization, including China (Han et al., 2020). The extent of China's food waste is alarming, with approximately 26.3% of the food produced for residential consumption wasted or lost each year. The food loss rate across the entire supply chain stands at 19.0% ± 5.8%, with the consumer segment being the largest source of waste, accounting for 7.3% ± 4.8% (Liu et al., 2013). The total estimated loss and waste amount to 422.56 million tons, representing about 22.37% of total food production (Jia et al., 2022), with wasteful practices continuing to rise (Zhang et al., 2022).

The causes of food waste are multifaceted, encompassing consumer behavior, food packaging design, and supply chain management (dos Santos et al., 2022). Food packaging, in particular, emerges as a significant driver of food waste, with research indicating that improvements in packaging design can mitigate this issue (Chan, 2022). As an indispensable component of the food supply chain, food packaging's primary functions are to extend shelf life and ensure food safety through physical protection, chemical barriers, microbial inhibition, and information dissemination (Salgado et al., 2021). Studies reveal that in developing countries, the volume of food waste attributable to packaging issues surpasses the quantity of food produced annually (Mao et al., 2008). Food packaging has consistently addressed the challenge of extending food longevity through more effective methods (Nair et al., 2023), aiming to mitigate food waste at its source rather than managing it through waste disposal strategies (Stangherlin and De Barcellos, 2018). This underscores the potential of food packaging design to influence and potentially reduce food waste behaviors. Therefore, investigating the mechanisms through which food packaging design impacts consumer behavior in reducing food waste is essential, as it can enhance the effectiveness of food packaging. Conversely, it can effectively mitigate environmental pollution (Versino et al., 2023). Furthermore, sustained guidance and influence can positively impact consumer behavior (Chu et al., 2020), thereby fostering the advancement of sustainable consumption (Coussy et al., 2013). It is acknowledged that consumers' perceptions and requirements regarding packaging vary across different regions, reflecting cultural and societal disparities, economic constraints (Zeng, 2021), and limitations imposed by infrastructure and environmental technologies in certain developing nations. Considering the overall trends in food packaging design, research into food packaging design holds significant importance in reducing food waste behaviors; however, the specific implementation strategies and packaging improvements must be adapted to account for environmental variations (Du Rietz Dahlström and Kremel, 2023). Consequently, through the strategic design of packaging elements, it is feasible to diminish food waste, lessen environmental impacts, and promote sustainable consumption practices.

This study aims to dissect the mechanisms through which food packaging influences consumer food waste behavior, identifying key design elements that encourage waste reduction. These elements include functional design aspects (e.g., preservation technologies, portion sizes; Yokokawa et al., 2019; Obersteiner et al., 2021), visual design (e.g., color schemes, graphic elements; Wang, 2013; Marques da Rosa et al., 2018), and informational design (e.g., consumption guidelines, environmental prompts; Lindh and Olsson, 2016; d'Astous and Labrecque, 2021). Through empirical research and longitudinal tracking, the study seeks to clarify the role of food packaging design in fostering thrifty consumer habits and promoting sustainable consumption models. The findings will provide actionable recommendations for businesses to optimize food packaging design and for governments to formulate relevant policies, thereby mitigating food waste at its source and contributing to global food security. The significance of this research is underscored by its multifaceted benefits. From a food safety perspective, reducing consumer-level food waste through packaging design enhances food resource utilization, alleviates the imbalance between food supply and demand, and strengthens national and global food security, thus safeguarding fundamental human needs (Barone and Aschemann-Witzel, 2022). Environmentally, food waste and packaging waste are intertwined, exacerbating resource depletion and environmental pollution. Inappropriate food packaging not only leads to material waste but also poses long-term hazards to ecosystems, such as soil and water bodies, due to its poor biodegradability (Georgakoudis et al., 2023). Scientific packaging design can extend food shelf life and reduce excessive packaging, thereby promoting ecologically sustainable development (Bakkaloglu and Arici, 2019). Economically, reducing food waste can lower production costs for businesses and improve economic efficiency (Martin-Rios et al., 2022). Socioculturally, food packaging, as a frequently encountered medium in daily life, can subtly integrate concepts such as food conservation and eco-friendly consumption through clever design, thereby guiding the public to develop healthy and civilized consumption habits. This, in turn, fosters a societal ethos of valuing food and embracing green living, contributing to the construction of a resource-efficient and environmentally friendly society (Suwandi et al., 2023).

Food packaging design must meet market demands while catering to consumers' desire for aesthetic enjoyment (Zhang, 2016). It can reflect the principles of fun and practicality, enhancing the consumption experience while highlighting the inherent value of the food itself (Biswas Majee et al., 2025). Therefore, conveying the concept of food conservation through packaging design can effectively influence consumer behavior regarding food. Relevant data shows that over 80% of consumers are willing to pay for environmentally friendly packaging (Ling et al., 2021). Consumers' recognition of green design and concepts in food packaging encourages enterprises and governments to prioritize this area, evident in the multifunctionality, sustainability, and recyclability of food packaging (Lindh and Olsson, 2016). Food packaging design can extend food safety and the lifespan of packaging (Pereira de Abreu et al., 2012), reflecting the ideals of conservation and environmental protection in both food and packaging (Opara and Mditshwa, 2013). Thus, education and the promotion of green ideas in food packaging design are particularly crucial (Xiao-b, 2014).

The research findings offer multifaceted contributions to practical applications. At the corporate level, identifying the factors influencing consumer food waste behavior through food packaging design enables businesses to reduce excessive packaging costs, develop lightweight and multifunctional packaging to minimize waste, and enhance market competitiveness. For consumers, incorporating designs that promote conservation and environmental awareness on packaging encourages rational consumption, fosters food-saving habits, and improves the consumer experience. Within the food packaging industry, the research provides a basis for establishing green packaging standards, driving the industry toward sustainability and multifunctionality. In terms of policy-making, the results offer data support for government implementation of green packaging incentive policies and regulation of excessive packaging, as well as providing a reference for integrating conservation and environmental concepts into social publicity and education policies. To elucidate the mechanism by which food packaging factors influence consumer behavior, we employ the Behavioral Reasoning Theory (BRT) to explain the motivations underlying consumers' rejection of food waste, predicting behavior through intention (Unal et al., 2024). We supplement this with the inclusion of consumer context, which allows for a more comprehensive consideration of various influencing factors in the actual purchase and consumption process. In the context of food packaging, considering specific factors in packaging design enables an accurate analysis of consumer behavioral intentions under specific packaging factor backgrounds, addressing the limitations of existing research that focuses on internal consumer factors while neglecting the contextual influence of external factors (Popovic et al., 2019). Specifically, BRT, by incorporating reasoning variables, compensates for the shortcomings of traditional behavioral theories (TPB), providing a more comprehensive explanation of behavioral intentions (Wirama and Wirama, 2024). Simultaneously, incorporating consumer context into the research scope makes the results more relevant to real-life consumption scenarios, where different packaging design factors have varying impacts on consumer behavior. This consideration of contextual factors aids in packaging design that better serves consumers' actual purchasing behavior. Utilizing BRT, we analyze the influence paths on consumers' rejection of food waste behavior through multi-level and multi-dimensional approaches. The study employs a two-stage modeling approach based on Partial Least Squares Structural Equation Modeling (PLS-SEM) to systematically analyze the second-order constructs of “Reasons for” and “Reasons against” (Xu et al., 2024). Specifically, in the first stage, path analysis is conducted on the observed variables corresponding to each first-order construct using the PLS-SEM algorithm, thereby obtaining latent variable information for the first-order constructs, which effectively refines and represents the core information contained within them. In the second stage, the latent variable information of the first-order constructs obtained in the first stage is used as measurement indicators for the second-order constructs, constructing a measurement model for the second-order constructs, thus realizing the modeling process from specific constructs.

## Literature review

### Behavior reasoning theory

According to Behavior Reasoning Theory (BRT), the rationality of behavior or the reasons behind actions is a more direct and critical factor influencing decision-making than traditional psychological variables such as values and attitudes (Westaby, 2005). Most scholars have conducted extensive and in-depth research on green low-carbon psychology and behavior using the Technology Acceptance Model (TAM), Theory of Reasoned Action (TRA), and Theory of Planned Behavior (TPB; Liu et al., 2017). These traditional models of green consumption have received substantial empirical support for the relationship between attitude and behavioral intention; however, they often overlook the rationality of behavior. Traditional theoretical models, predicated on the “rational consumer” assumption, posit that consumers engage in cost-benefit analyses to maximize utility, thereby illustrating the limitations of rational models in the study of user behavior (Jackson, 2005). Concurrently, consumer decision-making in related fields is often influenced by factors such as social norms, moral cognition, and status quo bias (Abhyankar, 2022). Furthermore, traditional models overlook the impact of contextual factors, such as habitual consumption and aesthetic preferences, thereby neglecting the rationalization of human behavior (Temizkan, 2022). Consequently, traditional theories struggle to explain the non-linear relationships between variables; for instance, the influence of attitudes on behavior may exhibit threshold effects due to the influence of other factors, which the traditional linear structures of models like TPB fail to capture (Velnampy and Achchuthan, 2016). The rationality of consumer behavior transcends the traditional rational assumptions, emphasizing the decision-making processes of consumers under conditions of bounded rationality, including the cognitive rationality of information acquisition and processing (Estrada, 2010), as well as contextual and habitual factors in decision-making (Kevin et al., 2024). Moreover, in the specific context of food packaging, numerous studies have explored the impact of factors such as the promotion of purchase intention through eco-friendly packaging and consumer preferences for recyclable and biodegradable materials (Kevin et al., 2024), as well as moderating factors like price and social influence (Chopde, 2024). Integrating these factors into the model is essential, highlighting the advantages of BRT in analyzing the interactions between variables. To investigate the impact of food packaging factors on consumer behavioral rationality, this study incorporates behavioral rationality into the traditional theoretical model. The BRT model offers unique advantages in analyzing behavioral rationality. Consequently, the BRT model is employed to analyze the underlying mechanisms of consumers' environmental behavioral intentions. Furthermore, values significantly influence consumers, exerting a long-term and fundamental effect. Traditional theoretical models, when discussing behavioral intentions, often focus on attitudes, subjective norms, and perceived behavioral control, yet overlook values as an antecedent variable. Therefore, this research integrates values into the discussion to examine how food packaging influences subsequent consumer attitudes, behavioral rationality, and behavioral intentions (Claudy and Peterson, 2013). Methodologically, this paper utilizes BRT to organically integrate consumer values, behavioral rationality, attitudes, and behavioral intentions, thereby exploring the intrinsic mechanisms of food packaging's impact on consumer behavior. Explaining consumer behavioral intentions through supporting/rejecting reasons is crucial, with values serving to predict behavioral rationality, attitudes, and behavioral intentions (Sahu et al., 2020).

Building upon the preceding discussion, this study centers on elucidating the mechanism by which food packaging influences consumers' behaviors in rejecting food waste. The research methodology is as follows: Grounded in the Behavior Reasoning Theory (BRT), with the incorporation of consumer context as a supplementary element, thereby addressing the limitations of traditional theories in overlooking external factors. This approach employs “reason for” and “reason against” variables to interpret behavioral motivations. Furthermore, it integrates specific aspects of food packaging, including functionality, visual design, and informational design, to construct a multi-dimensional influence pathway. The aim is to investigate their specific impacts on consumers' behavioral intentions and their actions in rejecting food waste.

### Food packaging design factors

Food packaging design factors represent a multidimensional and interdisciplinary field. It encompasses visual design, material science, environmental impacts, and consumer behavior. Multiple factors influence consumers' purchasing decisions. These include, but are not limited to, the packaging's visual attributes (Marques da Rosa et al., 2018; Ahmadi et al., 2013), functional properties (Yokokawa et al., 2019; Obersteiner et al., 2021), environmental attributes (Lindh and Olsson, 2016; d'Astous and Labrecque, 2021), and social connotations (Wang, 2013). The visual attributes of packaging, such as its color and shape, significantly impact consumers' product preferences and their impressions of a product's taste and health benefits (Marques da Rosa et al., 2018). Many elements of packaging design affect consumer behavior, including aspects such as its informational content, content protection, identification, smart features, geometric shape, eco-friendliness, durability, and color (Konstantoglou et al., 2020). The environmental attributes of food packaging include the use of recyclable materials, reduced resource consumption, and minimized environmental pollution (Wang and Sun, 2010). Meanwhile, the functional attributes of food packaging, including its durability, shelf-life extension capabilities, and controlled release technologies, are crucial in maintaining food quality and safety (Shima Jafarzadeh et al., 2023). Thus, food packaging designs contribute to consumers' confidence, ensuring that the materials do not contaminate or migrate to the food, as well as preventing physical damage (Helanto et al., 2019). Moreover, the eco-friendliness of food packaging influences not only purchasing intentions but also environmental intentions. In addition, consumers' awareness and understanding of eco-friendly packaging impact their purchasing decisions. Consumers' awareness and knowledge of eco-friendly packaging vary across different countries and regions. For instance, South African consumers possess limited knowledge about eco-friendly packaging and exhibit fewer environmentally friendly behaviors related to packaging (Scott and Vigar-Ellis, 2014). In Ghana, research also indicates that although consumers' awareness of environmental issues positively and significantly impacts their green purchasing decisions, the effect of green packaging itself on consumers' purchasing decisions is not significant (Rokka and Uusitalo, 2008). This finding suggests that consumers' sociodemographic characteristics can influence their awareness, behavior, and expectations regarding the environmental sustainability of food packaging. Factors such as their gender, age, and educational level affect consumers' perceptions and behaviors regarding the environmental sustainability of food packaging (Chirilli et al., 2022). To encourage consumers to adopt more sustainable food practices, packaging designers must consider visual attributes, functional properties, environmental characteristics, and social significance, while also ensuring that this information effectively reaches consumers. Additionally, they must account for cross-cultural differences and individual consumer traits to develop personalized and effective packaging design strategies (Lindh and Olsson, 2016; Borgman et al., 2018).

Meanwhile, poor packaging design negatively affects consumers' purchasing decisions, perceptions, and behaviors, which indirectly influences their food-saving habits. Excessive marketing claims on packaging, such as health and nutrition assertions, can mislead consumers, causing food to appear healthier than it actually is, leading to over-purchasing (Obersteiner et al., 2021). Furthermore, misleading information on packaging designs can cause consumers to develop misconceptions, resulting in food waste behaviors (Chandon, 2013; Cairo, 2015). Improper packaging sizes or difficult-to-open packages may prevent consumers from correctly storing or using their food, ultimately leading to premature spoilage and disposal (Boyce et al., 2008). Unreasonable packaging sizes can lead to consumer misunderstandings regarding the product capacity. If a container's shape is particularly large, it may be perceived as containing more of the product, even if the actual capacity is small (Valerie Folkes, 2004). The packaging dimensions can significantly impact consumers' perceptions of the product capacity; these may involve its volume, weight, and overall impressions, thus affecting consumers' perceptions of the amount of food purchased and contributing to food waste (Ordabayeva, 2013). Additionally, poorly functional packaging designs can increase food waste and exacerbate environmental issues (Konstantoglou et al., 2020). The functionality of packaging is often inadequate, failing to protect its contents and extend the shelf life of food, which consequently contributes to consumers' food waste behaviors (Ploom et al., 2020). Consumers' perceptions of the risks associated with packaging influence their decisions regarding food waste, where health consciousness and environmental awareness emerge as key factors. Poorly functional packaging tends to exacerbate food waste (Zeng, 2021). Research indicates that consumers' perceptions of the risks of packaging directly affect their food waste behaviors (Zeng, 2021). A lack of awareness among consumers regarding packaging design often results in misunderstandings about food's freshness and shelf life, leading to food waste (Obersteiner et al., 2021). Insufficient consumer awareness of food packaging safety can result in the neglect of both safety and environmental considerations while selecting packaging, further contributing to food waste (Kang and Wang, 2021). Overly complex or difficult-to-understand information on packaging diminishes consumers' engagement and attentiveness to this information, adversely affecting their purchasing decisions and food consumption and thus causing food waste (Mruk-Tomczak et al., 2019).

### Consumer waste behavior: factors associated with packaged food

The factors influencing consumer behavior regarding packaged food waste are multifaceted, with packaging design playing a crucial role in encouraging proper food consumption, waste reduction, and effective sorting and recycling of both food and packaging waste. As a critical intermediary between production and consumption, packaging design characteristics significantly influence consumer purchasing decisions. Furthermore, they indirectly affect the extent of food waste through storage practices and usage frequency. Research indicates that 60% of consumers try new products due to their packaging (Uddin et al., 2022), highlighting the impact of packaging design on consumer purchasing behavior. Packaging features also exert influence; studies show that visual and sensory cues affect consumer decision-making. For instance, red-orange is associated with “sweetness,” while blue-green suggests “health,” and angular designs enhance a modern aesthetic (Marques da Rosa et al., 2018). Transparent packaging enhances product authenticity, thereby influencing consumer visual experiences (Simmonds et al., 2017). Packaging quality is another significant factor in consumer decision-making. While some packaging utilizes recyclable materials, the cost often includes a premium (Zeng and Durif, 2019). Storage behavior is also linked to packaging quality; large-capacity packaging can lead to food waste and over-purchasing, while smaller packaging, though convenient, increases packaging costs, also incurring a premium (Wikström et al., 2019). Resealable packaging extends shelf life post-opening, reducing waste from spoilage, but it also increases packaging costs, again with a premium (Nemat et al., 2020a). This underscores that both the visual aspects and quality of packaging are critical factors influencing consumer decision-making. Consequently, visual attributes and packaging quality serve as communication channels, thereby encouraging consumers to conserve food and properly categorize food packaging waste (Nemat et al., 2020b). The functional and educational aspects of food packaging design, which can extend food shelf life and diminish food waste, are also critical determinants of consumer behavior (Almli et al., 2018).

Consumers' perceptions of and attitudes toward packaging largely determine whether they will reduce their food waste. In this context, consumers are unlikely to prioritize food packaging or reductions in food waste as the primary motivation in their purchasing decisions (Kapoor and Kumar, 2019). At the same time, consumers' inadequate knowledge about optimized packaging affects their ability to use these packages correctly at home (Obersteiner et al., 2021). Additionally, consumers' perceptions of green packaging also influence their decisions regarding food waste (Zeng, 2021). However, an insufficient understanding of the environmental impacts of food packaging may lead to choices that inadvertently negate consumers' eco-friendly intentions (Lindh and Olsson, 2016). The functionality of packaging plays a significant role in reducing food waste, as the functional information provided on food packaging can greatly impact consumers' choices regarding optimized packaging (Almli et al., 2018). Although the information conveyed through packaging design holds tremendous potential to reduce food waste, consumers still seldom pay proper attention to such information (Rokka and Uusitalo, 2008). Perceptions regarding packaging also reflect consumers' age, gender, and educational levels, as older consumers may generate more food waste (Hazuchova et al., 2020). Furthermore, consumers' perceptions and strategies regarding imperfect or secondary foods are essential in reducing food waste (Varese et al., 2022). Consumers' perceptions and behaviors play a crucial role in minimizing food waste. Additionally, consumers' awareness and actions regarding food waste issues are vital in achieving societal sustainability (Norton et al., 2022).

## Research hypotheses and models

### Research hypothesis

#### Values and consumer support and rejection reasons for food waste refusal

Values, representing stable psychological dispositions formed in daily life, shape an individual's perception of themselves and the world (Kesberg and Keller, 2018). Consumer values influence attitudes and behavioral intentions toward food packaging (Kumar et al., 2023). Drawing on Schwartz's dimensions of values, consumer values can be understood as openness to food packaging (Przymuszała et al., 2024). Research on attitudes and behavioral intentions toward food packaging highlights understanding and acceptance (Li et al., 2020). For instance, consumers' environmental values (environmental cognition acceptance) can influence their intention to purchase environmentally friendly packaging (Jha et al., 2023). Therefore, this study uses “consumer acceptance of food packaging” to represent consumer values, defined as an individual's willingness to engage with and understand new concepts. Acceptance is reflected in consumers' willingness to embrace and acknowledge novel packaging materials (e.g., bio-based materials, biodegradable plastics) and design concepts (e.g., circular packaging, environmental intentions). Research indicates that consumer inclusivity indirectly influences sustainable decision-making behaviors by affecting their environmental attitudes (Zhao et al., 2024). Food packaging, a persistent topic in the food industry, has packaging design factors that can satisfy consumers' food experience through protection and recommendation, especially in functional design, environmental concepts, and visual experience (Sumi and Swathilakshmi, 2023; Un-Noor et al., 2017). Consequently, for consumers with a high degree of openness to new things, excellent food packaging aligns with their values. Consumer acceptance of food packaging is both a manifestation of values and a behavioral driver, with its core being the construction of a trust bond between consumers and packaging through design innovation, information transparency, and cultural adaptation (d'Astous and Labrecque, 2021). Concurrently, decision theory (Fishburn, 1991) and attribution theory (Asanakutlu and Sahin, 2014) further affirm that values influence the reasons consumers support or reject specific behaviors. Thus, the development of behavioral rationality is not independent of beliefs and values; rather, values serve as the foundational basis for behavioral rationality (Henshel, 1971). Therefore, within the context of research on the rejection of food waste, this study posits that consumers' values (The acceptance of food packaging) impact their justifications for supporting or rejecting consumer behaviors that mitigate food waste. Based on this, the following hypothesis is proposed:

H1: Consumer values (The acceptance of food packaging) positively influence their support for reasons to refuse food waste.
H2: Consumer values (The acceptance of food packaging) negatively influence their rejection of reasons to refuse food waste.

#### Values and consumer attitudes toward food waste rejection

Research indicates that values are not only direct factors in the formation of attitudes but also serve as significant mechanisms that indirectly influence attitudes by providing reasons for support or rejection (Kristiansen and Zanna, 1988). Consequently, when consumers' values align with the values represented in food packaging, they are more likely to develop a positive attitude toward the product (Venkatesh and Davis, 2000), and at this juncture, behavioral justifications may not be activated (Afroz et al., 2015). In such cases, consumers tend to pursue psychological shortcuts and simplify personal information processing (Ju, 2015). Consumers who are more inclined to accept high-quality food packaging are the first to experience the design of food packaging, enjoy the food, and reject food waste, indicating their open-mindedness toward the rejection of food waste (Hinnüber et al., 2019). This value system encourages a positive attitude toward certain behaviors (Davis, 1989). Consumer attitudes toward the rejection of food waste may be formed through either intermediary or direct pathways. Therefore, when consumers develop their attitudes toward food waste rejection through direct pathways, values can influence attitudes without the need for behavioral rationalization (Afroz et al., 2015). Conversely, when attitudes are formed through intermediary pathways, values will influence attitudes indirectly via behavioral rationalization (Chaturvedi et al., 2023). Based on this, the following hypothesis is proposed:

H3: Consumers' values (inclusivity toward food packaging) positively influence their attitudes toward rejecting food waste.

#### Consumer support, opposition, and attitudes toward food waste rejection

Behavioral rationality refers to the specific justifications individuals employ to defend their expected behaviors, which are regarded as critical antecedent variables influencing attitudes and behavioral intentions (Chatzisarantis and Biddle, 1998). When individuals rationalize their anticipated actions, they often experience comfort, which, in turn, fosters a positive attitude toward those actions (Jones and Wirtz, 2006). Food packaging, as a significant factor facilitating consumer purchases and consumption of food, encourages consumer support for food waste rejection for several reasons, such as visual attributes in packaging design (Marques da Rosa et al., 2018; Ahmadi et al., 2013), functional attributes (Yokokawa et al., 2019; Obersteiner et al., 2021), environmental attributes (Lindh and Olsson, 2016; d'Astous and Labrecque, 2021) and social cognition (Wang, 2013). Conversely, there are also reasons for rejection, including over-information (Chandon, 2013; Cairo, 2015), regional cognitive disparities (Zeng, 2021; Obersteiner et al., 2021), and low functionality (Boyce et al., 2008; Ploom et al., 2020). These supportive and oppositional reasons further influence consumers' positive or negative attitudes toward rejecting food waste. Based on this, the following hypotheses are proposed:

H4: Consumers' supportive reasons for rejecting food waste positively affect their attitudes toward food conservation.
H5: Consumers' oppositional reasons for rejecting food waste negatively affect their attitudes toward food conservation.

#### Consumer support, rejection, and intentions regarding food waste behavior

Behavioral rationality possesses corrective and defensive mechanisms (Przeworski and Vreeland, 2002), yet prior theoretical models of behavior have not explained this aspect. Social psychology theories demonstrate that these mechanisms aid individuals in enhancing or preserving self-worth, allowing rationality to transcend attitudes in explaining behavioral occurrences (Malle, 1999). In other words, individuals feel more comfortable when they have justifications for correcting and defending their actions, even if their attitudes do not entirely align with their behavioral intentions. Consumers maintain a positive attitude toward green practices, but irrational packaging design creates a gap between green attitudes and behaviors. Consequently, this paper posits the following hypotheses:

H6: The reasons consumers support food waste rejection positively influence their behavior toward rejecting food waste.
H7: The reasons consumers oppose food waste rejection negatively influence their behavior toward rejecting food waste.

#### Consumer attitudes and behavioral intentions regarding food waste rejection

Although consumers may hold a positive attitude toward eco-friendly food, actual actions often experience the impact of the “value-action gap,” which refers to recognizing a problem without taking action (Zhuo et al., 2022). Research indicates that environmentally conscious consumers are more likely to take steps to reduce food waste (Chen, 2019). Specifically, feelings of responsibility and self-efficacy are crucial psychological factors that activate personal norms and further influence the intention to reduce food waste (Wang et al., 2022). Attitudes also directly and positively impact behavioral intentions. A study in West Sumatra found that attitudes significantly and positively influenced households' intentions to reduce food waste, which in turn strongly affected actual food waste behavior (Guchi and Syafrizal, 2022). Other research suggests that while consumers may be aware of the food waste issue, this awareness does not always translate into action, a phenomenon termed the “value-action gap” (Neff et al., 2015). Accordingly, this paper hypothesizes:

H8: Consumers' attitudes toward food waste rejection positively influence their intentions to engage in food waste rejection behavior.

### Theoretical model

**Figure 1:**
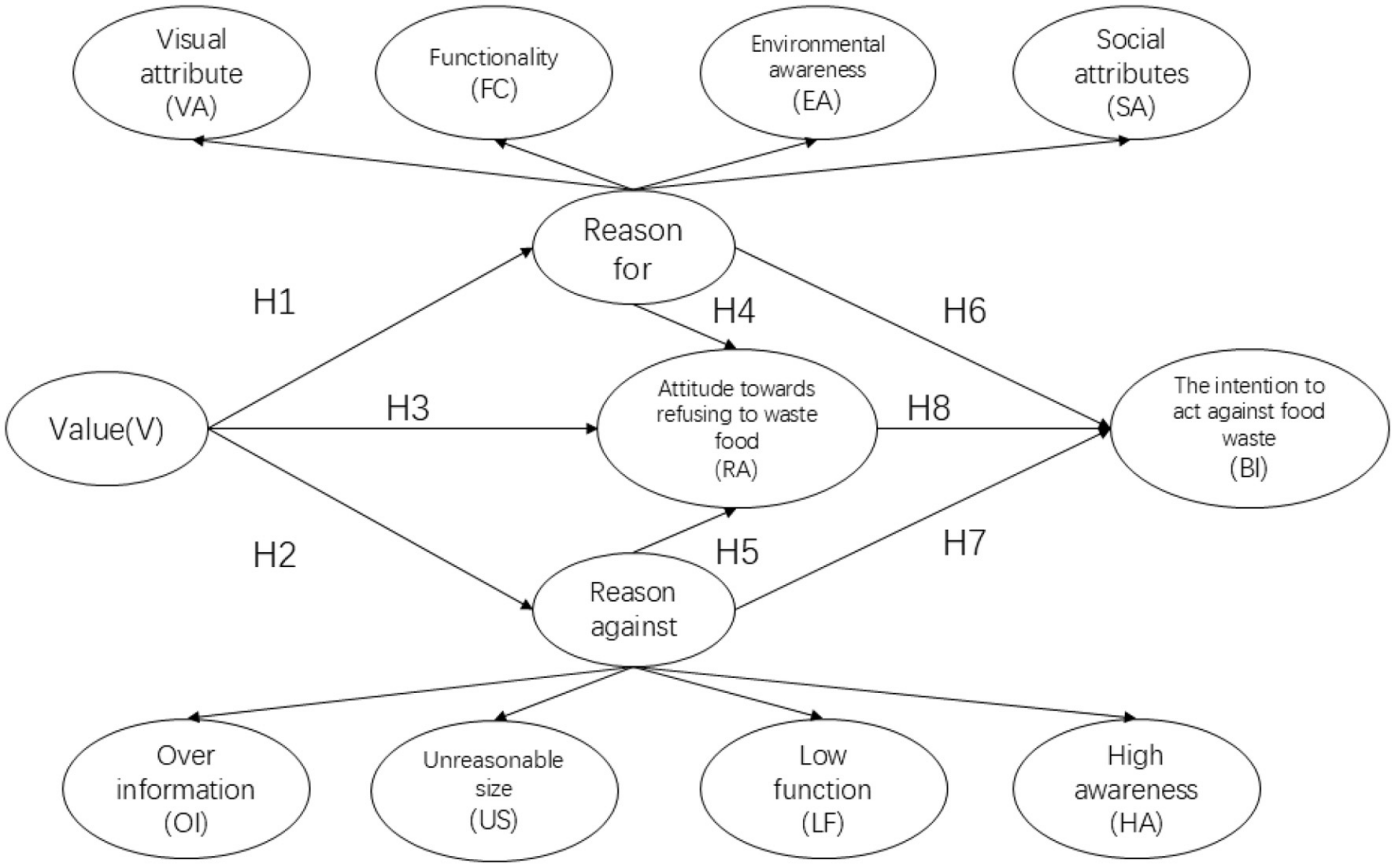
Structural equation model.

Based on the analysis above, we construct the following theoretical model, as shown in Figure 1.

### Questionnaire design

This study examines the influence of food packaging factors on consumer food waste reduction across eight categories, drawing on previous research and literature. Based on this foundation, a survey was designed to assess how various food packaging design elements and consumer values impact their support for or rejection of food waste. The aim is to validate the mechanisms and effects of food packaging design factors on consumers' cognitive attitudes and behavioral intentions.

From the perspective of cognitive attitudes, consumers' values—The acceptance of food packaging—affect their attitudes toward rejecting food waste, exhibiting both positive and negative influences. This is adapted from the viewpoints of Chan and Xiao regarding values (Chan, 2022; Xiao-b, 2014; V1–V3). Additionally, the eight types of food packaging design factors are analyzed for their impact on consumers' cognitive attitudes toward rejecting food waste, referencing the visual factors in food packaging design as studied by Wang (2013) and Marques da Rosa et al. (2018; VA1–VA3), functional attributes (Yokokawa et al., 2019; Obersteiner et al., 2021; FC1–FC3), environmental awareness factors (Lindh and Olsson, 2016; d'Astous and Labrecque, 2021; EA1–EA3), and social factors (Wang, 2013; SA1–SA3). These four dimensions are measured for their positive impact on consumers' rejection of wasteful behaviors.

Furthermore, regarding the negative impact of food packaging design factors on consumers' attitudes toward rejecting food waste, it includes four dimensions. Zeng believes that excessive marketing information, health information, and nutritional information in food packaging design can lead to consumer food waste behavior and affect food conservation (Zeng, 2021; OI1–OI3). Ploom and Pentus believe that Unreasonable size in food packaging design can lead to food waste behavior among consumers due to unreasonable packaging size, difficulty in opening, and size that is not easy to carry and store, thereby affecting food conservation (Ploom et al., 2020; US1–US3). Chandon believes that low functionality in food packaging design can lead to food waste behavior among consumers due to the inability to protect food, short shelf life, and unfavorable transportation, thereby affecting food conservation (Chandon, 2013; LF1–LF3). Obersteiner and Cociancig et al. believe that the differences in other social and cultural levels lead to High awareness of environmental protection in food packaging design. The phenomenon of protection leads to food freshness cognitive bias, shelf life cognitive bias and other information bias, which lead to food waste behavior and affect the item design of food conservation (Obersteiner et al., 2021; Ploom et al., 2020; HA1–HA3).

On the perspective of rejecting food waste, according to Kunda and Zhuo, Ren et al., it is believed that behavioral rationality can enhance individual awareness and self-worth, promote the generation of positive attitudes toward food conservation and environmental protection, but attitudes may not necessarily translate into action intentions (Zhuo et al., 2022; Kunda, 1990). On this perspective, item design is carried out to reduce food waste, protect the environment, reflect personal morality, and demonstrate the willingness to take practical actions for Attitude toward reusing to waste food (Neff et al., 2015; Fornell and Larcker, 1981; RA1–RA3).

From the perspective of behavioral intention, Wang, Li, Guchi and Anon's research on behavioral intention, including the influence of self-belonging and self-efficacy and attitude on behavior (Wang et al., 2022; Guchi and Syafrizal, 2022). This dimension examines the personal intention to act against food waste, the sense of self belonging and self-efficacy of behavior, and the implementation of specific behaviors (Neff et al., 2015; Gelman, 2006; BI1–BI3).

Based on the above analysis, the items for questionnaire design are shown in Table 1.

**Table 1:** Definition of variable operability and reference scales.

**Construct**		**Items**	**Source**
**First order**	**Second order**		
Values (The acceptance of food packaging) (V)		V1: You think you would rather accept different food packaging V2: You think that food packaging design has enhanced your perception V3: You recognize that the role of environmental education in food packaging design has brought about a change in your environmental attitude	Chan, 2022; Xiao-b, 2014
Reason for	Visual attribute (VA)	VA1: The graphic design content on the package conveys the idea of saving food VA2: The color design content on the package conveys the idea of saving food VA3: The text design content on the package conveys the idea of saving food	Wang, 2013; Marques da Rosa et al., 2018
	Functionality (FC)	FC1:The durability of the packaging function design conveys the idea of saving food FC2: Packaging function design prompts effective food shelf-life with less food waste FC3: The packaging design size is reasonable to pay attention to the storage state of food to reduce food waste	Yokokawa et al., 2019; Obersteiner et al., 2021
	Environmental awareness (EA)	EA1: The concept of saving food conveyed in the packaging design EA2: Packaging design materials design factors to reduce environmental pollution concept package EA3: Load design material use factor transfer reduce resource use transfer environmental ideas	Lindh and Olsson, 2016; d'Astous and Labrecque, 2021
	Social attribute (SA)	SA1: Focus on the recyclability of packaging materials in packaging design to reduce food waste SA2: Focus on reusability of packaging materials in packaging design to reduce food waste SA3: Pay attention to the idea of saving food transmitted in the packaging materials in the packaging design	Wang, 2013
Reason against	Over information (OI)	OI1: It is affected by too much marketing information in the packaging design and ignores the value of food resulting in waste OI2: It is affected by too much food health information in the packaging design and ignores the food value resulting in waste OI3: It is affected by too much nutritional information in the packaging design and ignores the food value resulting in waste	Zeng, 2021
	Unreasonable size (US)	US1: Food waste caused by unreasonable packaging size US2: Food waste is caused by the difficulty of packaging opening US3: Food waste caused by food packaging sizes that are not easy to carry and store	Ploom et al., 2020
	Low function (LF)	LF1: The packaging of food cannot protect food and cause food waste LF2: The short shelf life of food causes food waste LF3: The packaging of food is not conducive to transport, resulting in food waste	Chandon, 2013
	High awareness (HA)	HA1: The freshness of food mentioned in the packaging design is understood to be different resulting in food waste HA2: Insufficient understanding of food shelf-life mentioned in packaging design food waste HA3: Not understanding the information conveyed by packaging design leads to wrong understanding and food waste	Obersteiner et al., 2021; Ploom et al., 2020
Attitude toward refusing to waste food (RA)		RA1: Good food packaging has a positive impact on reducing food waste RA2: The rejection of food waste has a positive impact on environmental protection RA3: Refusing to waste food is a reflection of personal virtue RA4: Willingness to take practical actions to reduce food waste and reduce environmental damage	Wang et al., 2022; Guchi and Syafrizal, 2022
The intention to act against food waste (BI)		BI1: There is a strong desire to reduce food waste on environmental grounds BI2: Reducing food waste is good for your body and mind BI3: Promote food saving behavior to others	Neff et al., 2015; Gelman, 2006

### Informed consent

To facilitate data collection, an online questionnaire survey method was selected. Given the geographically dispersed consumer distribution discussed earlier, an online survey approach was deemed more appropriate than offline methods. Consequently, all respondents were recruited through the Wenjuanxing online platform. Furthermore, this study received ethical approval from the School of Fine Arts, Nanjing Normal University. An online informed consent form was presented prior to the commencement of the survey. Due to the online nature of participation, all participants provided their consent verbally. Respondents were directed to the survey questionnaire only after confirming their informed consent by selecting the “agree” option. Participants who selected the “disagree” option were excluded from the survey. This approach ensured the collection of authentic and comprehensive consumer survey data. All respondents were consumers who had purchased or used food packaging. Moreover, this study adhered to the Declaration of Helsinki and was approved by the Institutional Review Board of the School of Fine Arts, Nanjing Normal University (ID: NO. NNU SFA-E-2024-002, June 19, 2024). All participants provided informed consent, and their privacy was strictly protected. Personal information was kept confidential throughout the study. Participants were informed of their voluntary participation and their right to withdraw at any time. All respondents were adults, with no minors included.

## Empirical research

### Sample demographic characteristics

Data collection for this study utilized an online questionnaire format, distributed via online platforms to a broad demographic to ensure the sample's representativeness and mitigate regional biases, thereby enhancing the validity of the collected data. The survey was conducted between July and September 2024, utilizing the “Wenjuanxing” data survey platform to collect a total of 562 samples. After eliminating invalid samples, 513 valid samples were ultimately obtained, resulting in an effective rate of 91.3%. The reasons for sample exclusion were three-fold: (1) Duplicate submissions from the same IP address were consolidated, with only one retained as a valid sample; (2) Surveys completed in an excessively short timeframe were deemed invalid; (3) Samples containing sequences of repeated numerical entries were excluded. All items were assessed using a Likert scale, with scores ranging from 1 (strongly disagree) to 7 (strongly agree). Prior to participation, an online informed consent form was presented. Given the online nature of the survey, verbal consent was obtained from all respondents. Furthermore, participants were required to confirm their comprehension of the informed consent and indicate their agreement by selecting an affirmative option to access the questionnaire. At the same time, the respondents could only participate in the questionnaire survey once and could not participate in the questionnaire repeatedly, which ensured the accuracy of the questionnaire survey. Non-agreement resulted in the termination of the survey. This study did not involve minors, as they are considered to lack full behavioral capacity due to their developmental stage.

**Figure 2:**
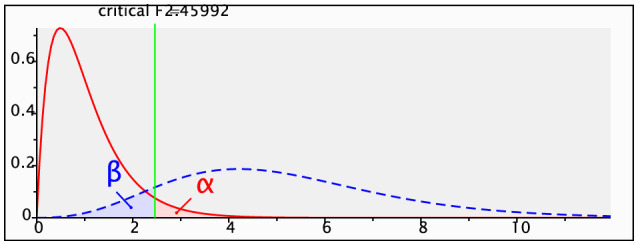
Sample size analysis chart of G*power.

The questionnaire comprised 34 items, yielding 513 valid responses. Statistical power analysis, conducted using G^*^Power, was performed for a linear multiple regression model, specifically focusing on the *R*^2^ deviation in a fixed model. As illustrated in Figure 2, the input parameters included an effect size *f*^2^ of 0.15, a significance level (α error probability) of 0.05, and a desired statistical power (1-β error probability) of 0.90, representing a 90% probability of correctly rejecting the null hypothesis. Consequently, under these parameters, G^*^Power calculated a required total sample size of 108. This sample size ensures sufficient statistical power to detect effects within the model, given the specified effect size and significance level. The actual statistical power was 0.9013736, slightly exceeding the target of 0.90, indicating that the sample size meets the study design requirements and supports the reliability of the findings. Given the effective sample size of 513, which substantially surpasses the minimum requirement of 108, the sample size is deemed adequate for the study.

Therefore, the data analysis proceeded based on this standard, primarily utilizing SPSS 22.0 and Smart-PLS 4.1 software. Descriptive statistical analysis of the sample demographic variables is presented in Table 2.

**Table 2:** Demographic profile of sample (*N* = 513).

**Sample**	**Category**	**Number**	**Percentage (%)**
Gender	Male	305	59.5
	Female	208	40.5
Age	18–24	89	17.3
	25–34	182	35.5
	35–44	115	22.4
	45–54	77	15
	Over 55	50	9.7
Occupation	Student	52	10.1
	Freelance or self-employed	60	11.7
	Public officials or public institutions	351	68.4
	Others	50	9.7
Overbuying packaged foods	Many	163	31.8
	Seldom	231	45
	Hardly	119	23.2
Dispose of excess packaged food	Continue to eat	144	28.1
	Drop	294	57.3
	Other	75	14.6
Sadness due to food disposal	Will	153	29.8
	Only a little	105	20.5
	Hardly	201	39.2
	Not at all	54	10.5

The survey encompassed 513 questionnaires, with sample regions strategically selected across 19 provinces, including Jiangsu, Fujian, Sichuan, Shandong, Anhui, Zhejiang, Hunan, Jiangxi, Hubei, Heilongjiang, and Guangdong, thereby demonstrating the comprehensiveness of the sample collection. Nevertheless, regional disparities in economic development influenced the distribution of consumers who had utilized or experienced food packaging. Notably, a significant proportion of respondents hailed from economically advanced regions such as Jiangsu, Fujian, Guangdong, and Zhejiang, indicating a heightened level of attention toward food packaging in more developed areas, thus validating the authenticity of the sample collection. The online survey participation comprised a slightly higher number of male respondents compared to female respondents, with the age distribution predominantly skewed toward young and middle-aged individuals. In terms of the occupational distribution, government workers and public institution staff accounted for the largest proportion, followed by freelancers and students, and other occupations accounted for approximately 10%. At the same time, according to the questionnaire, the proportion of respondents who overbought packaged food was as high as 86.8%, of which 31.8% of these respondents often overbought packaged food. When faced with a large amount of excess packaged food, 57.3% of respondents stated that they would directly discard it, and only 28.1% were willing to continue to eat it. Meanwhile, 71.9% of the 513 respondents would throw food away or use it for other purposes, and 49.7% did not feel sad or upset about food waste. This means that nearly half of the respondents were indifferent to food waste. In summary, the proportion of the sample that responded to each part of the questionnaire was reasonable, meeting the requirements of the research. According to the data on the respondents' activities related to buying packaged food, dealing with excessive packaged food, and discarding packaged food, it is necessary to study the influence of food packaging design factors on consumers' behavior in terms of rejecting food waste.

### Reliability and validity analysis

Reliability refers to the consistency or stability of measurement results. Research indicates that a Cronbach's α value between 0.7 and 0.9 is generally considered to reflect good internal consistency (Oviedo and Campo-Arias, 2005). The Cronbach's α values for each measurement variable, calculated using SPSS 22.0, were all greater than 0.7, while the overall Cronbach's α for the questionnaire exceeded 0.6 (Yin and Wei, 2012; Chan and Idris, 2017). The total correlations after item deletion were all above 0.5 (Yin and Wei, 2012), indicating that the constructs in this study possess good reliability. The variance inflation factor (VIF) serves as a metric for assessing multicollinearity among independent variables, quantifying the extent to which each predictor variable is linearly explained by others. A higher VIF value indicates a more severe multicollinearity issue (Willis and Perlack, 1978). Research suggests that a VIF exceeding 5 suggests the presence of some degree of multicollinearity within the measurement scale (Alhamami et al., 2024); ideally, the VIF should be below 3.3 (Bria et al., 2021). At this level, the correlation between predictor variables is low, resulting in more stable regression estimates, which is conducive to constructing robust analytical models. As shown in Table 3, the VIF values for all variables are below 3.3, indicating the absence of multicollinearity issues. Overall, the Cronbach's alpha coefficient (a reliability indicator) and the VIF values (a multicollinearity indicator) in Table 3 collectively demonstrate that the measurement scale used in this study has good reliability and does not exhibit multicollinearity issues, thus supporting the construction of a stable and reliable analytical model.

**Table 3:** Reliability analysis results (*N* = 513).

**Dimension**	**Items**	**Collinearity statistics VIF**	**Corrected item-to-total correlation**	**Cronbach's α if item deleted**	**Cronbach's α**
V	V1	1.830	0.673	0.712	0.806
	V2	1.711	0.644	0.743	
	V3	1.705	0.642	0.744	
VA	VA1	1.594	0.610	0.712	0.780
	VA2	1.591	0.609	0.712	
	VA3	1.675	0.635	0.684	
FC	FC1	1.666	0.632	0.745	0.802
	FC2	1.731	0.649	0.728	
	FC3	1.780	0.662	0.714	
EA	EA1	1.644	0.626	0.709	0.786
	EA2	1.634	0.623	0.712	
	EA3	1.645	0.626	0.708	
SA	SA1	1.728	0.649	0.719	0.798
	SA2	1.718	0.646	0.721	
	SA3	1.668	0.633	0.735	
OI	OI1	1.800	0.665	0.726	0.807
	OI2	1.685	0.638	0.755	
	OI3	1.788	0.662	0.729	
US	US1	1.656	0.627	0.706	0.785
	US2	1.705	0.643	0.689	
	US3	1.573	0.603	0.731	
LF	LF1	1.691	0.639	0.735	0.801
	LF2	1.697	0.640	0.733	
	LF3	1.762	0.658	0.715	
HA	HA1	1.502	0.578	0.713	0.768
	HA2	2.080	0.614	0.673	
	HA3	2.089	0.610	0.677	
RA	RA1	1.984	0.702	0.826	0.861
	RA2	2.144	0.730	0.814	
	RA3	2.067	0.718	0.819	
	RA4	1.871	0.682	0.834	
BI	BI1	1.632	0.622	0.741	0.796
	BI2	1.770	0.659	0.701	
	BI3	1.690	0.637	0.725	

Utilizing SPSS 22.0, the Kaiser-Meyer-Olkin (KMO) measure and Bartlett's test of sphericity were conducted. Relevant studies indicate that the KMO value serves as an indicator of the suitability of data for factor analysis, with a KMO value greater than 0.7 generally considered acceptable for this purpose (Campos et al., 2013; Yordanova and Krastev, 2018; Sara et al., 2020). Furthermore, a significance level of less than 0.05 in Bartlett's test suggests that the covariance matrix of the variables is not an identity matrix, indicating significant correlations among the variables, thus making them suitable for factor analysis (Wu and Wong, 2003; Dyer and Keating, 1980). As shown in Table 4, the KMO values for the variables ranged from 0.704 to 0.829, all exceeding the threshold of 0.7, and the significance of Bartlett's test was less than 0.05, confirming that all variables passed the Bartlett's test of sphericity. This establishes a solid foundation for the factor analysis of the data. Consequently, principal component analysis was employed for the factor analysis of the variables, revealing that each variable could extract a factor with an eigenvalue greater than 1, and the cumulative variance contribution of all variables exceeded 50%. This indicates that the factors identified in this study provide a robust explanation for the variables analyzed (Feng, 2012). Additionally, the commonalities for all items were greater than 0.5 (Liang and Khan, 2024), and the factor loadings were all above 0.6 (Elias et al., 2020), which are within reasonable limits. In summary, this study concludes that the survey results exhibit strong unidimensionality, indicating that there is no issue of common method bias in the data, as illustrated in Table 4.

**Table 4:** Exploratory factor analysis result (*N* = 513).

**Dimension**	**Items**	**KMO**	**Bartlett Sphere test**	**Factor loading**	**Commonality**	**Eigenvalue**	**Total variation explained (%)**
V	V1	0.712	0	0.861	0.742	2.160	72.013
	V2			0.843	0.710		
	V3			0.842	0.708		
VA	VA1	0.703	0	0.828	0.686	2.085	69.490
	VA2			0.828	0.685		
	VA3			0.845	0.714		
FC	FC1	0.711	0	0.836	0.700	2.149	71.628
	FC2			0.847	0.718		
	FC3			0.855	0.732		
EA	EA1	0.706	0	0.837	0.701	2.100	70.011
	EA2			0.835	0.698		
	EA3			0.838	0.702		
SA	SA1	0.711	0	0.848	0.720	2.138	71.281
	SA2			0.847	0.717		
	SA3			0.838	0.702		
OI	OI1	0.713	0	0.856	0.733	2.166	72.198
	OI2			0.838	0.703		
	OI3			0.854	0.730		
US	US1	0.704	0	0.839	0.703	2.099	69.961
	US2			0.848	0.719		
	US3			0.822	0.676		
LF	LF1	0.711	0	0.841	0.708	2.145	71.492
	LF2			0.842	0.709		
	LF3			0.853	0.728		
HA	HA1	0.697	0	0.811	0.657	2.049	68.315
	HA2			0.836	0.698		
	HA3			0.833	0.694		
RA	RA1	0.829	0	0.837	0.700	2.825	70.629
	RA2			0.856	0.732		
	RA3			0.848	0.718		
	RA4			0.822	0.675		
BI	BI1	0.708	0	0.831	0.691	2.131	71.031
	BI2			0.855	0.732		
	BI3			0.841	0.708		

When Cronbach's α and composite reliability exceed the 0.7 threshold, the scale is deemed acceptable, as indicated in Table 3, suggesting that the scale demonstrates satisfactory reliability. Validity analysis was conducted using Smart-PLS4.1, with validity assessed through Average Variance Extracted (AVE) and Composite Reliability (CR) values. As presented in Table 5, the CR values range from 0.768 to 0.862, and the AVE values range from 0.683 to 0.722. According to Fornell et al., both CR and AVE values are critical for assessing convergent validity, with CR values needing to be greater than 0.7, and 0.5 being the minimum acceptable AVE value (Fornell and Larcker, 1981; Churchill, 1979). The results in Table 5 indicate that all AVE values surpass the 0.5 threshold, and all CR values exceed the 0.7 threshold, confirming that all variables exhibit robust convergent validity.

**Table 5:** Convergent validity analysis results (*N* = 513).

**Parameters**	**Cronbach's alpha**	**Composite reliability (CR)**	**Average variance extracted (AVE)**
RA	0.861	0.862	0.706
EA	0.786	0.786	0.700
V	0.806	0.807	0.720
FC	0.802	0.802	0.716
HA	0.768	0.768	0.683
LF	0.801	0.801	0.715
OI	0.807	0.808	0.722
SA	0.799	0.799	0.713
BI	0.796	0.798	0.710
US	0.785	0.787	0.700
VA	0.780	0.781	0.695

0.5 is the lowest standard for AVE and CR value > 0.7.

As shown in Table 6, the outer loadings derived from the indicators of each construct in this study exceed the results of each additional construct's cross-loadings. It is noteworthy that cross-loadings are commonly employed as a preliminary step in assessing the discriminant validity of indicators (Leguina, 2015). Therefore, as indicated in Table 6, all variables demonstrate good discriminant validity.

**Table 6:** Discriminant validity: cross loading (*N* = 513).

	**RA**	**EA**	**V**	**FC**	**HA**	**LF**	**OI**	**SA**	**BI**	**US**	**VA**
BI1	0.659	0.648	0.612	0.618	0.618	0.62	0.662	0.641	**0.826**	0.653	0.624
BI2	0.705	0.688	0.695	0.687	0.700	0.701	0.703	0.692	**0.859**	0.689	0.687
BI3	0.688	0.668	0.681	0.656	0.689	0.650	0.682	0.689	**0.843**	0.659	0.676
EA1	0.677	**0.838**	0.655	0.668	0.648	0.685	0.667	0.669	0.657	0.680	0.666
EA2	0.676	**0.833**	0.685	0.659	0.641	0.668	0.648	0.650	0.667	0.644	0.648
EA3	0.681	**0.839**	0.661	0.672	0.648	0.648	0.656	0.655	0.667	0.667	0.683
FC1	0.707	0.654	0.663	**0.837**	0.676	0.671	0.680	0.697	0.636	0.656	0.670
FC2	0.682	0.671	0.682	**0.847**	0.664	0.664	0.688	0.682	0.668	0.665	0.684
FC3	0.702	0.697	0.682	**0.855**	0.668	0.687	0.689	0.694	0.668	0.689	0.671
HA1	0.643	0.620	0.630	0.644	**0.81**	0.633	0.659	0.627	0.641	0.637	0.631
HA2	0.677	0.643	0.646	0.659	**0.836**	0.643	0.683	0.642	0.680	0.662	0.660
HA3	0.666	0.650	0.672	0.657	**0.833**	0.673	0.644	0.666	0.650	0.671	0.652
LF1	0.692	0.675	0.662	0.672	0.674	**0.843**	0.688	0.678	0.659	0.676	0.698
LF2	0.693	0.663	0.674	0.662	0.652	**0.842**	0.682	0.676	0.659	0.665	0.676
LF3	0.694	0.684	0.678	0.686	0.667	**0.851**	0.670	0.686	0.661	0.660	0.679
OI1	0.683	0.681	0.683	0.684	0.656	0.698	**0.855**	0.690	0.694	0.696	0.680
OI2	0.687	0.634	0.677	0.669	0.668	0.666	**0.836**	0.658	0.661	0.661	0.675
OI3	0.743	0.685	0.692	0.712	0.716	0.687	**0.857**	0.707	0.709	0.707	0.730
RA1	**0.838**	0.683	0.662	0.701	0.670	0.701	0.679	0.705	0.689	0.687	0.690
RA2	**0.856**	0.707	0.691	0.700	0.688	0.692	0.726	0.679	0.699	0.699	0.713
RA3	**0.848**	0.703	0.691	0.700	0.662	0.700	0.706	0.696	0.684	0.696	0.704
RA4	**0.820**	0.629	0.658	0.668	0.672	0.662	0.676	0.652	0.658	0.661	0.673
SA1	0.714	0.682	0.684	0.704	0.670	0.694	0.682	**0.850**	0.689	0.704	0.678
SA2	0.675	0.664	0.659	0.686	0.650	0.672	0.688	**0.846**	0.675	0.669	0.666
SA3	0.669	0.647	0.655	0.677	0.657	0.670	0.674	**0.837**	0.662	0.656	0.665
US1	0.706	0.679	0.692	0.683	0.674	0.663	0.715	0.690	0.680	**0.842**	0.690
US2	0.706	0.684	0.701	0.693	0.701	0.679	0.700	0.682	0.679	**0.852**	0.688
US3	0.634	0.625	0.648	0.608	0.616	0.637	0.614	0.637	0.625	**0.815**	0.635
V1	0.717	0.697	**0.866**	0.707	0.707	0.691	0.717	0.681	0.698	0.734	0.711
V2	0.672	0.671	**0.843**	0.686	0.65	0.674	0.680	0.673	0.650	0.687	0.654
V3	0.656	0.660	**0.837**	0.638	0.642	0.655	0.651	0.654	0.655	0.648	0.662
VA1	0.648	0.663	0.663	0.641	0.629	0.667	0.648	0.636	0.644	0.665	**0.825**
VA2	0.735	0.681	0.668	0.689	0.673	0.689	0.711	0.678	0.663	0.682	**0.833**
VA3	0.684	0.645	0.662	0.664	0.657	0.667	0.686	0.668	0.660	0.661	**0.842**

The outer loadings results for the facets are represented by the bold numbers.

The Heterotrait-Monotrait Ratio (HTMT) is an index used to assess the discriminant validity of variables within a structural equation model. HTMT values below 0.85 are generally considered to indicate that the variables exhibit satisfactory discriminant validity (Henseler et al., 2015). It is essential to evaluate these metrics to mitigate potential multicollinearity issues arising from highly correlated constructs. The results indicate that all HTMT scores are within acceptable limits ( ≤ 0.85), with values ranging from 0.528 to 0.825 (Table 7). Therefore, it can be concluded that there is sufficient evidence to support the model's satisfactory discriminant validity in this study.

**Table 7:** Discriminant validity-Heterotrait ratio (*N* = 513).

**Latent variable**	**BI**	**EA**	**FC**	**HA**	**LF**	**OI**	**RA**	**SA**	**US**	**V**	**VA**
BI											
EA	0.739										
FC	0.748	0.553									
HA	0.762	0.564	0.577								
LF	0.753	0.528	0.579	0.581							
OI	0.737	0.517	0.568	0.586	0.540						
RA	0.796	0.612	0.572	0.596	0.569	0.583					
SA	0.758	0.542	0.572	0.562	0.544	0.567	0.611				
US	0.815	0.571	0.585	0.565	0.564	0.583	0.647	0.621			
V	0.825	0.588	0.572	0.595	0.554	0.575	0.620	0.645	0.616		
VA	0.741	0.547	0.567	0.578	0.579	0.581	0.583	0.594	0.565	0.594	

Discriminant validity refers to the differences among various latent variables. According to Fornell's recommendations, this can be verified by comparing the correlation coefficients between latent variables with the square root of the Average Variance Extracted (AVE). If the square root of the AVE is greater than the correlation coefficients of the variables, it indicates that the scale possesses good discriminant validity (Fornell and Larcker, 1981). As shown in Table 7, the square roots of the AVE for the latent variables are all greater than the correlation coefficients among the latent variables. Therefore, significant correlations exist among the main variables of this study, namely V, BI, EA, FC, HA, LF, OI, RA, SA, US, and VA, and the square roots of the AVE exceed the correlation coefficients between the variables. Moreover, the correlations of the exogenous structures are all less than 0.85 (Gelman, 2006), indicating good discriminant validity. According to Table 8, the square roots of the AVE for the latent variables are greater than the correlation coefficients among the latent variables, and the correlations of the exogenous structures are all less than 0.85. Thus, it can be concluded that all variables exhibit good discriminant validity.

**Table 8:** Correlation coefficient and average extraction variance (*N* = 513).

**Latent variable**	**RA**	**EA**	**V**	**FC**	**HA**	**LF**	**OI**	**SA**	**BI**	**US**	**VA**
RA	0.840										
EA	0.810	0.837									
V	0.804	0.797	0.849								
FC	0.824	0.796	0.799	0.846							
HA	0.801	0.772	0.786	0.791	0.827						
LF	0.820	0.797	0.794	0.796	0.786	0.846					
OI	0.829	0.785	0.805	0.810	0.801	0.804	0.850				
SA	0.813	0.787	0.789	0.816	0.781	0.804	0.807	0.844			
BI	0.812	0.793	0.787	0.777	0.795	0.781	0.810	0.800	0.843		
US	0.816	0.793	0.814	0.792	0.795	0.789	0.810	0.801	0.792	0.836	
VA	0.827	0.796	0.797	0.798	0.784	0.809	0.818	0.793	0.787	0.803	0.834

### Model testing

SRMR is one of the commonly used fit indices in Structural Equation Modeling (SEM) to evaluate the degree of fit between the model and the data. A smaller SRMR value indicates a better fit of the model to the data. Related research shows that SRMR performs well in handling ordered factor analysis models, particularly when data distribution is abnormal; in such cases, the testing results of SRMR are more reliable. An SRMR value less than 0.08 indicates a very high fit of the model to the data (Ximénez et al., 2022). Furthermore, an NFI greater than 0.8 reflects a high fit of the model; the closer the value is to 1, the stronger the model fit, signifying that the model can accurately capture the structures and relationships within the data (Gowen et al., 2011). As shown in Table 9, the SRMR value of 0.058 is below the threshold of 0.08, indicating a good model fit. Additionally, the NFI value of 0.964 is greater than 0.8, indicating a high model fit.

**Table 9:** Model fit measures.

**Common indices**	**SRMR**	**NFI**
Criteria	< 0.08	>0.8
Values	0.058	0.964

### Path hypothesis analysis

Finally, the hypotheses were tested using PLS-SEM, and path coefficients were calculated by employing the Bootstrap method with 5,000 resamples, as shown in Table 10. The *P*-values for the second-order model hypotheses were all found to be less than 0.05, indicating statistical significance. Therefore, the second-order model hypotheses were validated. As presented in Table 8, the statistical results demonstrate that all eight second-order model path relationships are significant, indicating that visual attributes, social attributes, environmental attributes, and functional attributes serve as positive reasons for consumers to reject food waste, while excessive information, low functionality, unreasonable sizes, and overly high cognitive load are identified as negative reasons for consumers' rejection of food waste.

**Table 10:** Second order model path relationship test.

**Path**	**β**	**STDEV**	***T* statistics**	***P* values**	**Decision**
Reason for -> VA	0.918	0.008	120.048	^***^	Accept
Reason for -> EA	0.916	0.008	117.035	^***^	Accept
Reason for -> FC	0.927	0.006	148.660	^***^	Accept
Reason for -> SA	0.922	0.006	144.546	^***^	Accept
Reason against -> US	0.921	0.006	147.488	^***^	Accept
Reason against -> HA	0.916	0.007	128.205	^***^	Accept
Reason against -> LF	0.918	0.007	135.311	^***^	Accept
Reason against -> OI	0.929	0.006	164.033	^***^	Accept

Significance of *P* value: ^***^P ≤ 0.001, ^**^P ≤ 0.01, ^*^P ≤ 0.05.

The analysis of the path coefficients from Table 11 indicates that values (The acceptance of food packaging) significantly influence the reasons for support (*T* = 84.088, *P* < 0.01) and the reasons for rejection (*T* = 83.473, *P* < 0.01), thereby providing support for hypotheses H1 and H2. However, the results regarding attitudes toward food conservation (*T* = 1.215, *P* > 0.01) are not significant, leading to the rejection of hypothesis H3. Furthermore, the reasons for support significantly affect attitudes toward rejecting food waste (*T* = 8.302, *P* < 0.01) and the behavioral intention to reject food waste (*T* = 4.372, *P* < 0.01), confirming hypotheses H4 and H6. Similarly, the reasons for rejection significantly influence consumers' attitudes toward rejecting food waste (*T* = 7.333, *P* < 0.01) and their behavioral intention to reject food waste (*T* = 6.131, *P* < 0.01), thus supporting hypotheses H5 and H7. Lastly, consumers' attitudes toward rejecting food waste significantly impact their behavioral intention to reject food waste (*T* = 1.758, *P* < 0.05), confirming hypothesis H8.

**Table 11:** Hypothesis model path relationship test.

**Hypothesis**	**Path**	**β**	**STDEV**	***T* statistics**	***P* values**	**Decision**
H1	Value (V) -> Reason for	0.863	0.010	84.088	^***^	Accept
H2	Value (V) -> Reason against	0.868	0.010	83.437	^***^	Accept
H3	Value (V) -> RA	0.048	0.040	1.215	ns, 0.224	Not accept
H4	Reason for -> RA	0.454	0.055	8.302	^***^	Accept
H5	Reason against -> RA	0.419	0.057	7.333	^***^	Accept
H6	Reason for -> BI	0.332	0.076	4.372	^***^	Accept
H7	Reason against -> BI	0.432	0.070	6.131	^***^	Accept
H8	RA -> BI	0.134	0.055	2.431	^*^, 0.015	Accept

Significance of *P* value: ^***^P ≤ 0.001, ^**^P ≤ 0.01, ^*^P ≤ 0.05.

**Figure 3:**
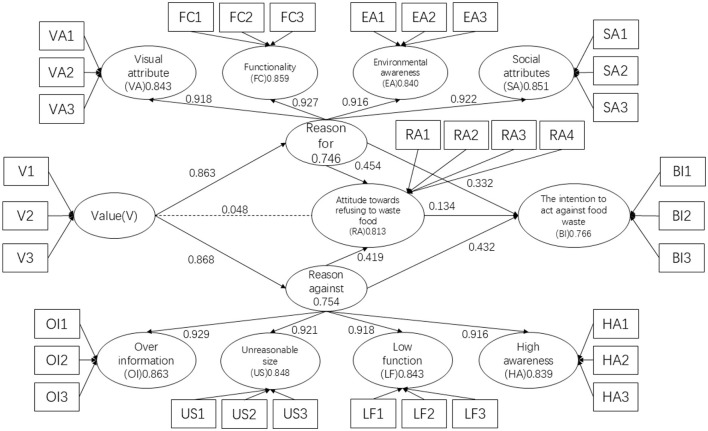
Hypothesis test results.

The results of hypothesis testing are shown in the above table, as shown in Figure 3.

## Discussion

This study employs the Behavior Reasoning Theory (BRT) as its foundation, incorporating consumer context to elucidate behavioral motivations through the variables of “reason for” and “reason against.” By integrating specific elements of food packaging, including its functionality, visual design, and informational aspects, this research delves into the correlation between food packaging factors and consumers' rejection of food waste. Furthermore, it explores consumers' attitudes toward food waste reduction and their behavioral intentions to conserve food. The subsequent discussion will unfold in accordance with the research model's structural sequence, integrating the validation of hypotheses and the presentation of results derived from the structural equation model. Key findings of this research are as follows:

Regarding the second-order model, the study confirms that visual, functional, environmental, and social attributes of food packaging design positively influence consumers' attitudes toward rejecting food waste. Conversely, excessive information, inappropriate dimensions, low functionality, and high cognitive load in food packaging design significantly and negatively impact consumers' attitudes toward rejecting food waste. From a data perspective, among the positive attributes, functional attributes (FC; T: 148.66, *P* < 0.01) exhibit a more significant influence on attitudes toward rejecting food waste than the other three attributes. Social attributes (SA; *T*: 144.546, *P* < 0.01) follow, demonstrating a more significant impact than visual attributes (VA; *T*: 120.048, *P* < 0.01) and environmental attributes (EA; *T*: 117.035, *P* < 0.01). This conclusion aligns with the findings of Aschemann-Witzel et al. 92015), Chen and Jai (2018), and Kim et al. (2020), indicating that consumers prioritize the functional and social attributes of food packaging. Among the negative attributes, excessive information (OI; *T*: 164.033, *P* < 0.01) has the most substantial impact on consumers' food waste behavior, representing the most significant factor contributing to food waste. This is followed by inappropriate dimensions (US; *T*: 147.488, *P* < 0.01) and low functionality (LF; *T*: 135.311, *P* < 0.01), with inappropriate dimensions showing greater significance than low functionality. The factor of high information cognitive load (HA; *T*: 128.205, *P* < 0.01) exhibits slightly lower significance. The significant negative impact of information overload on consumers aligns with Hunteret al.'s (2024) findings. Furthermore, the significance of information overload and unreasonable package size exceeding that of low functionality corroborates Chandon's (2013) assertions. Overall, the detrimental aspects of food packaging exert a more significant influence on consumers than the beneficial aspects. The *T*-values for rejection reasons are consistently higher than those for supporting reasons, indicating a lower tolerance among consumers for food packaging. Inferior food packaging has a more pronounced effect on consumers' attitudes and behaviors regarding food waste. While superior food packaging can alter consumer attitudes and behaviors toward rejecting food waste, its impact is less substantial than the detrimental effects of poor packaging. Consequently, research on the influence of food packaging factors on consumer attitudes and behaviors reveals that negative factors have a greater impact than positive factors, highlighting the more prominent harm caused by inferior packaging.

Regarding hypothesis validation, the confirmation of H1 and H2 indicates that consumer values, specifically their perception of food packaging inclusivity, significantly influence both supporting and refuting arguments. This suggests that superior food packaging design substantially impacts consumers' rationale for supporting food conservation, while inferior packaging significantly affects their reasons for rejecting such practices. This demonstrates that food packaging design directly influences consumers' justifications for either supporting or rejecting behaviors related to food waste. Conversely, the rejection of H3 implies that consumers in the context of food packaging cannot solely rely on their values to form attitudes against food waste; they also require information derived from food packaging design elements. This finding diverges from Isabel Schäufele's assertion that consumer values, such as health and environmental consciousness, directly influence purchasing attitudes and intentions, highlighting the differential impact of consumer values on attitudes toward various behavioral intentions (Schäufele and Janssen, 2021). This observation underscores that while consumer values significantly affect justifications related to food packaging, they do not directly influence attitudes against food waste, and their impact varies across different behavioral intentions.

The validation of hypotheses H4 and H6 confirms that the four second-order models within food packaging exert a significant positive influence on consumers' supporting reasons, attitudes toward rejecting waste, and behavioral intentions. The significance of this influence is more pronounced on attitudes than on behavioral intentions, indicating that food packaging is more effective in shaping consumers' attitudes toward rejecting food waste. Hypotheses H5 and H7 are also validated, demonstrating that the four second-order models in food packaging have a significant negative impact on consumers' rejection reasons, attitudes toward food conservation, and behavioral intentions. The significance of this negative impact is nearly equivalent on both attitudes and behavioral intentions, suggesting that poor food packaging design has a comparable detrimental effect on consumers' attitudes and behavioral intentions. A comparative analysis reveals that the significance of supporting reasons on attitudes (*T*: 8.302) is greater than that of rejection reasons on attitudes (*T*: 7.333), implying that superior food packaging design is more likely to foster positive consumer attitudes. Furthermore, the significance of supporting reasons on behavioral intentions (*T*: 4.372) is less than that of rejection reasons on attitudes (*T*: 6.131), indicating that inferior food packaging has a more substantial negative impact on consumers' waste-averse behaviors than superior packaging has a positive impact. Finally, the validation of H8 confirms the significant influence of attitudes on behavioral intentions. While both food packaging design factors and attitudes significantly affect behavior, consistent with the findings of Jeżewska-Zychowicz and Jeznach (2015) and Govindappa and Radha (2013), which suggest that packaging factors and consumer attitudes interact to influence consumer behavior, this study further reveals that the significance of attitudes toward rejecting food waste on food conservation behavior is considerably less than the significance of supporting/rejection reasons on food conservation behavior. This suggests that consumers are more readily influenced by food packaging factors in their waste-averse behaviors. Based on the conclusions drawn from hypotheses H4 to H8, consumer values significantly influence the reasons for supporting or rejecting food packaging. However, they do not directly impact attitudes toward rejecting food waste, and their effects on different behavioral intention attitudes vary. This suggests that, within the context of food packaging, the objective characteristics of the packaging itself exert a greater influence on consumers' behavioral intentions than their subjective attitudes.

## Theoretical contributions and practical implications

This study examines the hypothesized relationships between structural paths and the validation of model-based path relationships regarding consumers' attitudes and behavioral intentions to reject food waste, influenced by food packaging design factors. The research aims to simulate consumers' intentions to conserve food by integrating food packaging design elements with consumers' rejection of food waste and cognitive attitudes. The validation indicates that the quality of food packaging design factors directly impacts consumers' reasons for support and rejection, thereby affecting their attitudes and experiences related to food, ultimately influencing their stance against food waste and hindering their behavioral intentions to reject it.

### Theoretical contributions

Through empirical analysis, this study reconfirms the influence of consumer values and food packaging design elements on consumer emotions, clarifying their crucial roles in shaping consumers' conservation attitudes and behavioral intentions, thereby further enriching and refining the theoretical framework of the relationship between consumer behavior and food packaging design. Simultaneously, a novel theoretical model is innovatively constructed to deeply explore the relationship paths between food packaging design elements and consumers' attitudes and behavioral intentions toward rejecting food waste, providing a new perspective and research direction for the theoretical development in the field of food packaging design, which has significant reference value for the theoretical research of food packaging design. Furthermore, the research not only analyzes the impact of food packaging design elements and consumer values on consumers' supporting/rejecting reasons, attitudes, and behavioral intentions but, more importantly, builds a theoretical framework for future analysis of food packaging design guiding consumer behavioral intentions, which is conducive to the in-depth development of subsequent related research in this field.

### Practical contributions

The research findings offer valuable insights for the advancement of design elements in food packaging, encompassing visual appeal, functionality, environmental sustainability, and social impact. These insights assist designers in understanding the roles of various elements in influencing consumer attitudes and behavioral intentions, thereby facilitating the optimization of food packaging design. Furthermore, the research outcomes delineate key priorities for the future development of food packaging, emphasizing the importance of enhancing consumers' attitudes and behavioral intentions toward food conservation. This directs designers to prioritize the strategic application and integration of packaging design elements, tailoring designs to the specific attributes of different food products. This approach aims to capture consumer attention and encourage food-saving behaviors. The study also identifies unfavorable aspects of food packaging design from the consumer's perspective, offering recommendations to mitigate these issues. This contributes to a more positive consumer experience, strengthens the perceived value of food conservation, and ultimately fosters the formation of intentions to conserve food. In addition, the research underscores the significance of innovative food packaging design, encouraging designers to leverage design effects and objectives to reshape consumer perceptions of food waste. This, in turn, promotes the adoption and practice of food-saving behaviors among consumers, providing direct guidance for food packaging design practices.

Consequently, this study has yielded significant findings at both theoretical and practical levels. Theoretically, it addresses research gaps, refines the existing theoretical framework, and establishes a robust foundation for future academic investigations. Practically, it offers clear guidance and actionable strategies for the food packaging design industry, assisting the sector in promoting consumer behaviors conducive to food conservation through optimized packaging design. The research outcomes hold immediate practical significance and provide substantial support and positive direction for the future advancement of food packaging design, both in terms of theoretical research and practical innovation, thereby contributing to the evolution of the food packaging field toward greater scientific rigor and enhanced efficiency.

## Conclusions and suggestions

### Conclusions

This study delves into the correlation between consumer values and food packaging design elements, with a specific focus on their impact on consumers' value attitudes and behavioral intentions regarding food waste reduction. Positive food packaging factors encompass visual appeal, functional utility, environmental sustainability, and social significance. Conversely, negative factors include information overload, diminished functionality, inappropriate sizing, and elevated cognitive costs. By integrating these factors, a comprehensive research model was constructed, effectively capturing the behavioral shifts experienced by consumers after interacting with food packaging. This model offers significant guidance for the advancement of food packaging design.

Empirical data validation robustly demonstrates a significant association between food packaging design elements and consumers' attitudes and behavioral intentions. Notably, consumers exhibit a high degree of endorsement for the conservation and environmental principles embodied in food packaging design, coupled with a sustained intention to act accordingly. This finding not only elucidates consumers' value preferences in food packaging selection but also provides direction for the future development of food packaging design.

The findings of this study offer significant insights and practical value for packaging designers. They can leverage these results to gain a deeper understanding of consumer needs and expectations, enabling more precise articulation of design concepts. This approach ensures that food packaging effectively addresses consumer requirements on a functional level while also resonating with their values. By optimizing food packaging design, we anticipate fostering a positive consumer attitude and behavior toward reducing food waste, thereby contributing to resource conservation and environmental protection. Future research should build upon these findings, exploring how food packaging design can further influence consumer behavior and how innovation in this area can be achieved across diverse cultural contexts.

### Future research suggestions

The present study acknowledges certain limitations inherent in its methodology. The primary reliance on online questionnaires for data collection, while facilitating rapid and large-scale information gathering, introduces potential biases in sample representativeness. Specifically, the demographic profile of online survey respondents may skew toward younger age groups, leading to an underrepresentation of both adolescent and elderly populations. Adolescents, as the future drivers of societal change, are a critical demographic for cultivating food conservation awareness, given their formative consumption patterns. Simultaneously, the burgeoning elderly population in China, influenced by established habits and potentially slower adoption of new concepts, warrants significant attention in consumer behavior research.

To address these limitations, future research should adopt a mixed-methods approach. Quantitatively, this could involve optimizing online questionnaire distribution channels, collaborating with schools and community centers to target specific demographics, and expanding the sample size of adolescent and elderly participants. Furthermore, incorporating offline surveys, such as paper-based questionnaires administered in senior activity centers and schools, would enhance sample diversity. Qualitatively, in-depth exploration of the underlying motivations and behavioral drivers related to food conservation among adolescents and the elderly is essential. For instance, in-depth interviews and discussions can be employed to ascertain the conservation education they received within their familial and scholastic environments, as well as the influence of peer groups on their food conservation attitudes. For the elderly demographic, an exploration of traditional life experiences and memories of material scarcity can illuminate how these factors have shaped their current consumption habits and the underlying reasons for their resistance to emerging conservation concepts. Furthermore, organizing discussions on food conservation topics among adolescents and senior citizens from diverse age groups and backgrounds can facilitate the observation of idea exchange and consensus-building within these groups, thereby yielding richer and more profound insights.

Integrating large-scale data statistical analysis from quantitative research with the in-depth insights from qualitative research allows for a comprehensive understanding of the overall trends and disparities in food conservation awareness and behavior across different groups at a macro level. Simultaneously, it enables an in-depth analysis of the complex psychological and social factors underlying individual behaviors at a micro level. This comprehensive approach provides a more complete and thorough understanding of the research subjects, offering a more robust theoretical and data foundation for refining related research, thereby enhancing the practical guidance value and social significance of the research findings.

## Data Availability

The original contributions presented in the study are publicly available. This data can be found here: https://doi.org/10.6084/m9.figshare.28908881.v2.
